# The need to study rural cancer outcome disparities at the local level: a retrospective cohort study in Kansas and Missouri

**DOI:** 10.1186/s12889-021-12190-w

**Published:** 2021-11-24

**Authors:** Jeffrey A. Thompson, Lynn Chollet-Hinton, John Keighley, Audrey Chang, Dinesh Pal Mudaranthakam, David Streeter, Jinxiang Hu, Michele Park, Byron Gajewski

**Affiliations:** 1 Department of Biostatistics & Data Science, University of Kansas Medical Center, Mail Stop 1026, 3901 Rainbow Blvd, Kansas City, KS 66160 USA; 2 University of Kansas Cancer Center, University of Kansas Medical Center, 3901 Rainbow Blvd, Kansas City, KS 66160 USA; 3 Department of Biostatistics and Bioinformatics, Rollins School of Public Health, Emory University, 1518 Clifton Rd. NE, Atlanta, GA 30322 USA

**Keywords:** Cancer, Rurality, Healthcare disparities, Socioeconomic factors

## Abstract

**Background:**

Rural residence is commonly thought to be a risk factor for poor cancer outcomes. However, a number of studies have reported seemingly conflicting information regarding cancer outcome disparities with respect to rural residence, with some suggesting that the disparity is not present and others providing inconsistent evidence that either urban or rural residence is associated with poorer outcomes. We suggest a simple explanation for these seeming contradictions: namely that rural cancer outcome disparities are related to factors that occur differentially at a local level, such as environmental exposures, lack of access to care or screening, and socioeconomic factors, which differ by type of cancer.

**Methods:**

We conducted a retrospective cohort study examining ten cancers treated at the University of Kansas Medical Center from 2011 to 2018, with individuals from either rural or urban residences. We defined urban residences as those in a county with a U.S. Department of Agriculture Urban Influence Code (UIC) of 1 or 2, with all other residences defines a rural. Inverse probability of treatment weighting was used to create a pseudo-sample balanced for covariates deemed likely to affect the outcomes modeled with cumulative link and weighted Cox-proportional hazards models.

**Results:**

We found that rural residence is not a simple risk factor but rather appears to play a complex role in cancer outcome disparities. Specifically, rural residence is associated with higher stage at diagnosis and increased survival hazards for colon cancer but decreased risk for lung cancer compared to urban residence.

**Conclusion:**

Many cancers are affected by unique social and environmental factors that may vary between rural and urban residents, such as access to care, diet, and lifestyle. Our results show that rurality can increase or decrease risk, depending on cancer site, which suggests the need to consider the factors connected to rurality that influence this complex pattern. Thus, we argue that such disparities must be studied at the local level to identify and design appropriate interventions to improve cancer outcomes.

**Supplementary Information:**

The online version contains supplementary material available at 10.1186/s12889-021-12190-w.

## Background

Recently, there has been great interest in studying cancer outcome disparities among various groups, such as rural vs. urban residents [1–5]. Rural residence has often been presented as a risk factor for poor cancer outcomes [1]. However, numerous studies of this topic have resulted in seemingly conflicting results [2, 3]. Although some of these differences are likely due to disparate research methods and the challenge of defining rurality at the individual level, many of these inconsistencies may be attributable to using rurality as a surrogate for a range of risk factors that likely vary by region and disease. A simple variable like rural residence encapsulates a number of potential explanatory factors, such as access to care, lifestyle, environmental exposure, and other socioeconomic factors. Several of these factors are potentially modifiable through targeted interventions, and there is a great need to understand disparities that may influence cancer outcomes in specific rural and urban populations. Therefore, we investigated cancer outcome disparities as they relate to rural and urban residence among patients treated at the University of Kansas Medical Center from 2011 to 2018. We aimed to evaluate disparities in our patient population and identify intervention targets to enhance our institution’s current cancer prevention and control efforts. Given that many of these factors may vary by cancer and not just area of residence, we investigated the ten most common cancer types treated at our institution.

A common thought has been that rural residence may relate to reduced access to care [4, 6, 7]. In fact, a national study of cancer clinical trials found that when access to care was uniform, rural and urban subjects had similar outcomes [5]. If access to care is a major driver of cancer outcome disparities, rural residents may present with a higher stage at diagnosis. However, previous work has provided seemingly contradictory results, with studies reporting higher or lower stage of colorectal and lung cancer at diagnosis in different national and regional study populations. Although the literature may contain apparently contradictory results, we believe they reflect the complexity of questions surrounding urban rural disparities, caused by the interplay of issues such as transportation, local environment, diet, job opportunities, and other socioeconomic factors creating disparities that affect urban and rural residents differently in different places.

While rural cancer outcome disparities have been reported, rural residence may be a complex surrogate for the set of factors truly influencing risk of later cancer stage or higher mortality. We suggest these disparities may reveal the specific needs of populations served by particular institutions or locales. In this study, we examined the association between rural residence status and two cancer outcomes, mortality and initial stage at diagnosis, in ten cancers. By using inverse probability of treatment weighting (IPTW) [8] to create a synthetic balanced sample for nonmodifiable confounding factors likely to influence these outcomes, we aim to estimate disparities in rural and urban regions that may reflect potentially modifiable factors underlying differences in mortality. In this study, “treatment”, in the context of IPTW, refers to rural or urban residency status. Our study seeks to evaluate the complex role of rurality in cancer outcomes, guiding future interventions to reduce outcome disparities in the population served by the University of Kansas Medical Center. The University of Kansas Medical Center is located in Kansas City, Kansas a large urban center, which is also close to Kansas City, Missouri. Thus, it is accessible to a bistate population including approximately 25% rural residents. However, this unique position likely contributes a diverse mix of factors that are more complex than simple rural/urban residency. We suggest similar studies are necessary throughout the country to adequately identify the needs of both urban and rural populations and improve cancer outcomes.

## Methods

### Retrospective cohort and supplemental datasets

The aim of our study was to determine if rural residence affects tumor stage at diagnosis or survival for the 10 most common cancers seen at the University of Kansas Cancer Center. Our retrospective cohort was comprised of a total of 43,374 cancer cases treated at KU Cancer Center from the years 2011–2018 identified from the Curated Cancer Clinical Outcomes Database (C3OD) of the KU Cancer Center [9], which is a joint effort of the University of Kansas Medical Center (KUMC) and the University of Kansas Health System. We used the ICD-O-3/WHO 2008 site recode values to define cancer types, though all codes for colon cancer, rectal cancer, and oral and pharyngeal cancer were combined to create colon, rectal, and oral cancer groups. We then restricted our patient population to include only those diagnosed with the 10 most common cancers treated at KUMC (*N* = 26,133 cases): breast, lung, prostate, melanoma, oral, kidney, colon, non-Hodgkin’s lymphoma - nodal, bladder, and corpus uteri.

For each case, we obtained data on rurality of residence, sex, age, race, insurance status, marriage status, class of case, whether the case was a first malignancy, AJCC stage, vital status, and days from diagnosis until last follow-up. Rurality of residence was classified using 2013 Urban Influence Codes (UICs) developed by the United States Department of Agriculture, with urban counties defined as having UICs of 1 or 2 (metropolitan counties) and all other counties defined as rural. Urban and rural counties were grouped together to create a dichotomous variable (simply either urban or rural). Class of case was simplified to “Diagnosed at KUMC” or “Diagnosed Elsewhere”. Insurance status was simplified to “Public”, “Other”, or “No Insurance”, where “Public” includes Medicaid, Medicare, TRICARE, Military, Veterans Affairs, or Indian/Public Health Service and “Other” includes private or other insurance. Marriage status was defined as “Married”, “Single”, “Separated or Divorced”, or “Widowed”; a small number of cases listed as unmarried (as opposed to single) or unknown were removed (*N* = 362). AJCC stage was simplified to stage 0, 1, 2, 3, or 4, and stage 0 and 1 were grouped due to the low numbers of stage 0 cases for many cancers. Additional exclusions included male breast cancer cases (*N* = 37) and all cases with any other missing data (*N* = 1327) for a total of 24,407 cases, which are described in Table 1. A list of the site recodes used to define these 10 cancers is given in Table S1.

**Table 1:** Demographics

Variable	Level	Rural	Urban	***p***-value
		*n* = 3014	*n* = 21,393	
Race (%)	White	2862 (95.0)	18,752 (87.7)	< 0.001
	Black	50 (1.7)	1935 (9.0)	
	Other	102 (3.4)	706 (3.3)	
Insurance (%)	Other	1348 (44.7)	10,191 (47.6)	0.001
	No Insurance	49 (1.6)	234 (1.1)	
	Public	1617 (53.6)	10,968 (51.3)	
Marriage Status (%)	Married	2082 (69.1)	13,501 (63.1)	< 0.001
	Never Married	420 (13.9)	3387 (15.8)	
	Separated or Divorced	231 (7.7)	2286 (10.7)	
	Widowed	281 (9.3)	2219 (10.4)	
Class of Case (%)	Diagnosed at KUMC	845 (28.0)	6923 (32.4)	< 0.001
	Diagnosed Elsewhere	2169 (72.0)	14,470 (67.6)	
First Malignancy (%)	One Primary Ever	2417 (80.2)	16,748 (78.3)	0.018
	More Than One Primary	597 (19.8)	4645 (21.7)	
Age (median [IQR])		63.00 [55.00, 70.00]	63.00 [55.00, 71.00]	0.014
Cancer (%)	Oral	295 (9.8)	1394 (6.5)	< 0.001
	Colon	186 (6.2)	1289 (6.0)	
	Lung	354 (11.7)	3485 (16.3)	
	Melanoma	253 (8.4)	1499 (7.0)	
	Breast	601 (19.9)	6925 (32.4)	
	Corpus Uteri	144 (4.8)	1065 (5.0)	
	Prostate	515 (17.1)	2286 (10.7)	
	Bladder	166 (5.5)	927 (4.3)	
	Kidney	341 (11.3)	1309 (6.1)	
	NHL - Nodal	159 (5.3)	1214 (5.7)	
Stage	No stage	603 (20.0)	3898 (18.2)	< 0.001
	0/1	904 (30.0)	7485 (35.0)	
	2	582 (19.3)	3541 (16.6)	
	3	414 (13.7)	2740 (12.8)	
	4	511 (17.0)	3729 (17.4)	

In order to examine the differential effects of rural residence on socioeconomic factors, county level data for all U.S. counties on median household income was obtained from the U.S. Census Small Area Income and Poverty Estimates (SAIPE) Program for the year 2018 and on educational attainment from the U.S. Census American Community Survey for 2018. In order to study the differential effects of rural residence on binge drinking, data were obtained from a study by the Institute for Health Metrics and Evaluation [10]; unfortunately, no data for the state of Wyoming were available.

### Analysis

All analyses were carried out in the R statistical environment (v4.0.2). Our main analyses of the effect of rural residence on tumor stage at diagnosis and overall survival were carried out on our dataset comprised of our local catchment area (including patients from both Kansas and Missouri) and the results were contrasted with results observed previously at the national level. As an example of modifiable risk factors, which rurality may serve as a surrogate for, we examined differences in median household income, educational attainment, and binge drinking at both the national level and in our local catchment area. To focus the causal inference of rurality, we created a pseudo-sample balanced for likely confounders, including gender, race, insurance, marriage status, class of case, first malignancy status, and age, using the inverse probability of treatment weighting approach via propensity scores [11]. We did not include tumor stage at diagnosis in the weighting for the survival analysis, because stage is plausibly on the causal path from rurality of residence to cancer mortality and thus is a potential mediator of interest rather than a confounder. If place of residence leads to a higher tumor stage, which subsequently leads to a greater risk of mortality, we wanted to estimate the association between area of residence and stage to inform future interventions for higher risk patients. Stage was treated as an ordinal outcome, and we analyzed the effect of rurality on stage at diagnosis using IPTW cumulative link models, using the ordinal package for R. We analyzed the effect of rurality on overall survival using weighted Cox proportional hazards models (with weights truncated at the 99th percentile) and IPTW case weighting using the coxphw package for R, as well as Kaplan-Meier survival curves using the survival package for R. Two types of weighting were used: the weighted Cox proportional hazards models to address issues of non-proportional hazards and estimate an average hazard ratio, and the IPTW case weighting to create the balanced pseudo sample [12]. To provide additional context, we also provide models stratified by gender (although we considered doing the same for race, the numbers were too small to make this viable). Additional analyses of socioeconomic status and binge drinking at the national level used geographically weighted regression with the GWmodel package for R [13] and at the local level using simple linear regression. Tests of spatial nonstationarity used the F3 test [14]. Other regression model tests used Wald tests. All tests were based on a significance level of 0.05.

## Results

### Stage at diagnosis

**Fig. 1:**
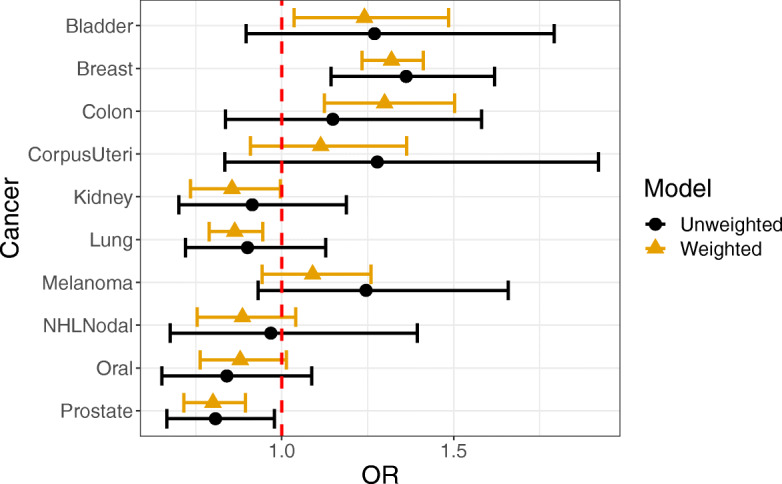
Cancer stage in rural vs. urban residents. The figure shows odds-ratios, with 95% confidence intervals, for the odds of being at a higher stage for rural vs. urban residents. We show results for both IPTW weighted and unweighted models. For the IPTW weighted models, rural residence had a significant effect on stage for bladder, breast, colon, kidney, lung, and prostate cancer. Rural residency was associated with increased stage in bladder, breast, and colon cancer but decreased stage for kidney, lung, and prostate cancer

Using IPTW cumulative link models of tumor stage (as an ordinal outcome), we estimated the odds ratio for rural vs. urban residence for each cancer site while controlling for differences in patient characteristics. We found that area of residence was significantly associated with stage at diagnosis for many cancers. In breast, colon, and bladder cancer, rural residents had significantly higher odds of being diagnosed with a higher stage cancer than urban residents. In contrast, rural residents had lower odds of being diagnosed with a higher stage lung, prostate, or kidney cancer than urban residents. Results with and without IPTW weighting are shown in Fig. 1. Odds-ratios, confidence intervals, and *p*-values can be found in Table S2 and Table S3. We found the IPTW approach was important to these results. Without weighting, colon, lung, kidney, and bladder cancer were not statistically significant, although the estimate of the effect was nevertheless close between weighted and unweighted in most cases. Results stratified by gender can be found in Fig. S1 and Fig. S2 and Table S4 and Table S5.

### Overall survival

**Fig. 2:**
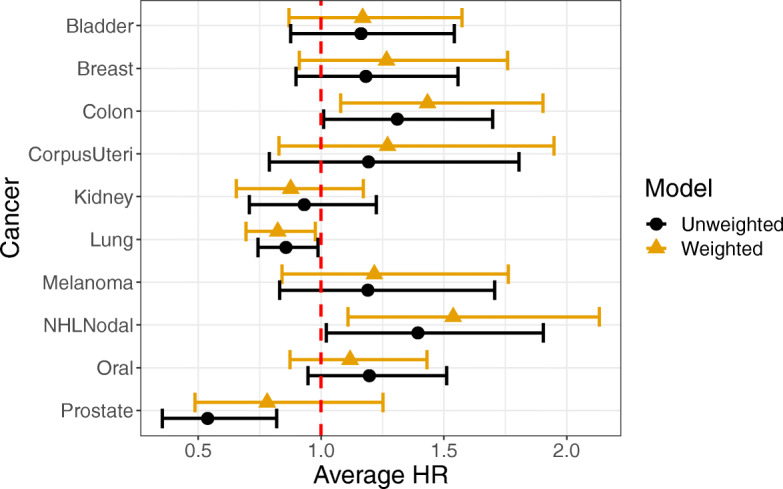
Hazard ratios for rural vs. urban residents. The figure shows average hazard-ratios, with 95% confidence intervals, for the hazards of rural vs. urban residents with respect to overall survival. We show results for both IPTW weighted and unweighted models. For the IPTW weighted models, rural residence had a significant effect on overall survival for colon cancer, lung cancer, and non-Hodgkin’s lymphoma. Rural residency was associated with increased hazards in colon cancer and non-Hodgkin’s lymphoma, but decreased hazards in lung cancer

Using weighted Cox proportional hazards models with IPTW case weighting, we estimated the hazard ratios for rural vs. urban residence and mortality for each cancer. Results with and without IPTW weighting are shown in Fig. 2. Hazard ratios, confidence intervals, and *p*-values can be found in Table S6 and Table S7. In many cases, we found the hazards were increased for rural vs. urban dwellers, with statistically significant results for colon and non-Hodgkin’s lymphoma (NHL – nodal). For some cancers, the hazard was lower in rural dwellers, with statistically significant results for lung cancer. In most cases, the effect of the IPTW case weighting was not substantial, although we observed a significant effect of rurality on survival for prostate cancer in the unweighted model that was attenuated and non-significant after IPTW weighting. Results stratified by gender are available in Fig. S3 and Fig. S4 and Table S8 and Table S9.

Therefore, after creating a pseudo-sample balanced for differences in patient characteristics that can affect cancer survival, it appears rural residence is associated with increased mortality for some cancers while demonstrating a protective effect for other cancers.

**Fig. 3:**
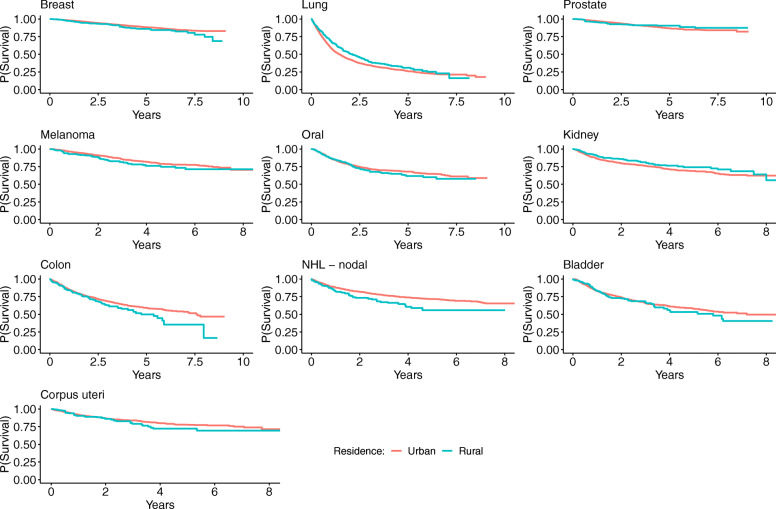
IPTW weighted Kaplan-Meier plots for the cancers in this study

To provide a different perspective, we also show the IPTW weighted Kaplan-Meier survival curves in Fig. 3.

### Stage as a mediator of overall survival

**Fig. 4:**
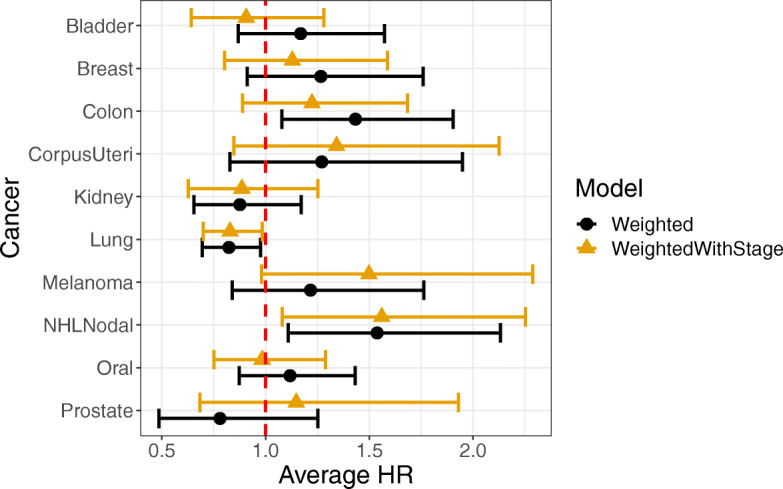
Hazard ratios for rural vs. urban residents after adjusting for stage. The figure shows average hazard-ratios, with 95% confidence intervals, for the hazards of rural vs. urban residents with respect to overall survival. We show results for IPTW weighted models with or without an additional covariate for stage. Previously, rural residence had a significant effect on hazards for colon and lung cancer as well as non-Hodgkin’s lymphoma. After adjusting for stage, the effect for colon cancer was no longer significant

We did not include stage in the IPTW weighting due to the potential for it to be a mediator of the effect of rural residence on survival. However, it is also plausible it might act as a mediator only for certain cancers, for example, in colon cancer due to early screening efforts that could differentially impact the two populations. Due to the lack of mediation analysis methods for dealing with a potential ordinal mediator, we did not perform a full mediation analysis. However, we did model overall survival using stage as a covariate for rural residence status in the IPTW models. In most cases, we found the inclusion of stage did not change any conclusions (Fig. 4). Our previous models showed a significant effect of rural residence on survival for colon, lung, and non-Hodgkin’s lymphoma. After controlling for stage, the effect of rural residence was reduced for colon cancer and became non-significant, an expected finding if stage is a mediator of the effect of rural residence on colon cancer survival. No differences were observed for lung cancer and non-Hodgkin’s lymphoma after adjusting for stage.

### Socioeconomic status

The effect of rural residency on cancer outcomes may vary among localities with respect to socioeconomic status [15]. However, most measures of socioeconomic status were not available to us at the individual cancer case level, so we could not address this hypothesis directly. Nevertheless, we were able to evaluate differences in county-level measures with respect to rurality that may be relevant for individual-level associations. Thus, we examined the impact of rural residency on median household income (MHI) and education (percentage of population 25 and over with at least a bachelor’s degree), both nationally and locally, as both income and education have previously been linked to cancer mortality [16, 17]. We performed geographically weighted regressions of each outcome and its association with rural residence. We tested these models for spatial non-stationarity using the F3 test (i.e., a test for evidence of a geographical relationship between the variables). For both MHI and bachelor’s degree the results were statistically significant at *p* < 0.001. Therefore, there is evidence that the relationship between rural residence and MHI or bachelor’s degree varies geographically. Furthermore, for both variables, we found a globally statistically significant relationship between rural residence and MHI and education at *p* < 0.001. The geographically weighted regression estimates a separate coefficient for each county. The distribution of these coefficients is shown in Table 2.

**Table 2:** Distribution of coefficients for the effect of rural residence on MHI or education

Response	Min	1stQuartile	Median	3rdQuartile	Max
MHI	−3.458	−1.387	−1.110	−0.825	0.054
Proportion with bachelor’s degree	−20.775	−9.327	−7.418	−5.177	2.346

The global estimate for the effect of rural residence on MHI was -$12,498 (*p* < 0.001). That is, rural residents have an MHI of approximately $12,498 less than urban residents on average. We also performed a simple linear regression for the effect of rural residence on MHI only in the counties in our catchment area. The estimate of the effect of rural residence on MHI locally was -$9384 (*p* < 0.001). The global estimate for the effect of rural residence on the percentage of people aged 25 or over with at least a bachelor’s degree was − 7.838% (*p* < 0.001), with a local effect in our catchment area of − 5.872% (*p* < 0.001). Therefore, in both cases, the rural disparities were somewhat less locally than the national average.

**Fig. 5:**
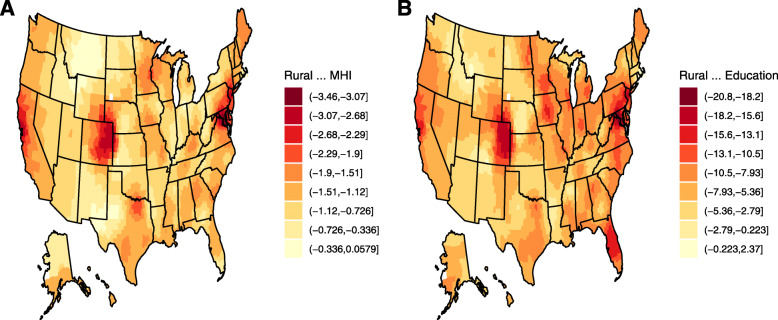
The association of rural residence with median household income and education nationally. The figure shows the distribution of geographically weighted coefficient estimates for the effect of rural residence on A) median household income and B) the percentage of a county’s population aged 25 and over with at least a bachelor’s degree. These plots show a similar pattern for how rural residence affects socioeconomic measures. We created this plot using the ggplot2 package of the open-source R statistical environment

We visualized the distribution of estimates for the effect of rural residence on MHI and education in Fig. 5. The coefficients were discretized into a series of bins for the purposes of visualization. The figures show that there are similar patterns for how rural residence affects MHI and education throughout the country.

Finally, to provide a simple summary result, we calculated the mean of median household incomes in urban vs. rural counties among all counties represented in our data. The mean of median household incomes in rural counties was $48,207 compared to $60,705 in urban counties, or a difference of $12,498. When restricting to the counties served by our institution, we found that that the mean MHI in rural counties was $50,183 compared to $59,567 in urban counties, or a difference of $9384. Therefore, the disparity in MHI is a little less in our region than it is nationally.

Similarly, the mean percentage of people age 25 or over with at least a bachelor’s degree in rural counties nationally is 18.7%, while for urban areas it is 26.5% (note that this is not equivalent to the percentage of people with a bachelor’s degree, due to the uneven distribution of the population), for a difference of 7.8%. In the counties included in this study, the mean proportion of people with a bachelor’s degree in rural counties is 21.4%, while for urban counties it is 27.3%, for a difference of 5.9%. So again, the disparity is smaller than what is seen nationally.

### Differential exposures

**Fig. 6:**
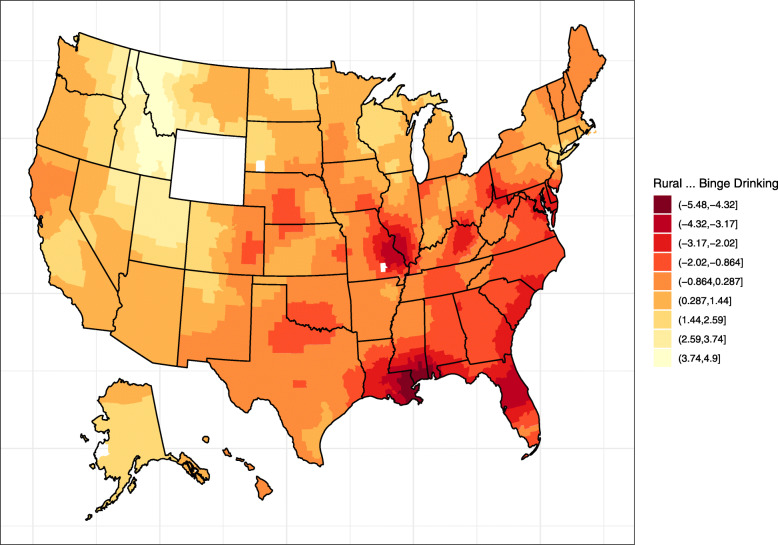
The association of rural residence with binge drinking nationally. The figure shows the distribution of geographically weighted coefficient estimates for the effect of rural residence on the percentage of residents in a county who engage in binge drinking. Data for Wyoming were not available. We created this plot using the ggplot2 package of the open-source R statistical environment

We considered whether rural residents might face different exposures associated with cancer risk depending on their geographic region. Again, we performed a geographically weighted regression of the effect of rural residence on the proportion of residents in a county who reported binge use of alcohol. We tested the model for spatial non-stationarity using the F3 test, which was significant at *p* < 0.001, indicating the effect of rural residence on binge drinking varies geographically. The global test for an effect was statistically significant, suggesting that rural residence is associated with binge drinking (*p* = 0.020). The distribution of coefficient estimates is shown in Table 3 and visualized in Fig. 6.

**Table 3:** Distribution of coefficients for the effect of rural residence on binge drinking

Response	Min	1stQuartile	Median	3rdQuartile	Max
Percentage of population binge drinking	−5.470	−1.302	−0.372	0.587	4.893

The global estimate for the effect of rural vs. urban residence on binge drinking was 0.440 (*p* = 0.020) nationally and − 0.625 (*p* < 0.001) within the counties in our catchment area. Thus, rural residence is associated with almost a half percent higher rate of binge drinking nationally while inversely associated with over a half percent lower rate of binge drinking locally.

## Discussion

In this study, we examined cancer outcome disparities in overall survival and stage at diagnosis based on a case’s rurality of residence. In line with other studies utilizing the Nebraska cancer registry [18] and the California Cancer Registry [19], we found rural residence was associated with higher stage of colon cancer at diagnosis as well as worse survival compared to urban residents. Notably, this is at odds with a national study using SEER registry data that showed urban residence was associated with higher stage colon cancer at diagnosis [20]. Others have reported differences in cancer outcomes according to rurality across different geographic regions [21], suggesting inconsistent associations between rural residence and cancer outcomes may be indicative of diverse risk factor profiles that differentially impact cancer mortality within rural regions. Indeed, we observed rurality may serve as both a risk and protective factor for mortality depending on cancer type, which may reflect differences in screening patterns, access to care, lifestyle, and socioeconomic factors that vary according to cancer site and local community characteristics. Taken together, these findings highlight the need for studies at the local level that consider differences across cancer types as well as factors underlying the definition of rural residence.

For breast, colon, and bladder cancers we found cancers were more likely to be diagnosed at a higher stage in rural compared to urban residents, with significantly higher risk of mortality for colon cancer and NHL - nodal. This is notable, as breast and colon cancers are commonly screened preventively among people who do not exhibit clinical symptoms. Our finding may indicate improved access to screening in urban residents served by KUMC compared to rural residents, emphasizing the importance of ongoing work by members of the KU Cancer Center to identify local at-risk communities and improve screening for underserved populations [22–24].

For lung, prostate, and kidney cancer, we found cancers were likely to be diagnosed at a lower stage for rural compared to urban residents. Previous work has suggested heavy alcohol consumption may be associated with increased prostate cancer mortality [25]. While we were unable to evaluate alcohol use within our population of cancer cases, we found conflicting associations between rural residence and binge drinking at the national and local levels. Specifically, binge drinking was more prevalent among rural residents compared to urban residents nationally, while with urban residents more likely to engage in binge drinking for the counties in our catchment area. This suggests the potential for poorer outcomes for alcohol-associated cancers in urban areas within our population. In contrast with a national study reporting reduced lung cancer survival in rural residents [26], we observed significantly lower lung cancer mortality in our local population of rural compared to urban residents. This reaffirms the importance of identifying potential factors driving differences in cancer outcomes with respect to rurality, ultimately informing the development of appropriate education, resources, and screening interventions tailored to specific, higher risk communities. Also, while proximity to care is a likely issue for rural residences, our work suggests a complex association with later stage at diagnosis given that lung, prostate, and kidney cancer were more likely to be diagnosed at a later stage among urban residents. This underlines the need to better understand how factors influence access to care beyond proximity in rural settings, including number and specialization of care providers, treatment availability, transportation, and cost of care. Indeed, we did find that some indicators of socio-economic status vary geographically with respect to rural residence.

Given that urbanicity or rurality was statistically significantly tied to both stage at diagnosis and overall survival for lung and colon cancers, developing interventions for these two cancers within our catchment area may be a priority. In particular, our results suggest stage may be a mediator of the effect of rural residence for overall survival in colon cancer. Indeed, efforts are already underway to reduce barriers to colon cancer screening across Kansas and Missouri. Given other possible environmental factors that we did not evaluate but may be related to these outcomes, future work examining the impacts of drinking habits, diet, physical activity, and air pollution exposures may reveal novel interventions to improve cancer prevention and control efforts in our population.

Our findings should be considered in light of some limitations. In our study, we were unable to analyze socioeconomic or risk factor exposure data at the individual level. However, we did find that disparities in two socioeconomic factors were smaller in our population compared to the national average, highlighting that these disparities may differ regionally. Follow-up studies conducted among individuals may reveal disparities in income and wealth gaps across our catchment area at the individual level. Another limitation was the lack of methods for fully performing mediation analysis with ordinal data, although we did find a suggestion of mediation by stage for the effect of rural residence on overall survival in colon cancer. Furthermore, given our cancer case population was institutionally sampled, we did not evaluate differences in cancer incidence according to rurality, and we were limited in our ability to conduct cancer-specific survival studies given that cause of death data were not available for most cases. We also note that median household income is not a complete measure of socioeconomic status, and it does not capture the cost of living. Many areas with a high cost of living also have a higher MHI. However, the same is not true for education. Thus, the similar results observed for education and MHI suggest differential disparities for socioeconomic status related to rural and urban residence depending on the region of the country.

Finally, our definition of rural residence was determined at the county-level, and we were unable to examine whether more localized definitions of rural residence based on zip code or specific community may further clarify the complex role of rurality in cancer mortality across cancer types that our study reveals.

In summary, our study presents strong evidence that rural vs. urban disparities in cancer outcomes exist in our patient population and are complex, representing both a risk factor and protective effect for mortality depending on cancer type. Considering the inconsistent results of other studies, our results suggest rural cancer outcome disparities are a local phenomenon that must be studied at the local level in order to intervene in the most efficacious fashion.

## Conclusions

In this study, we evaluated the associations between rurality, stage at diagnosis, and cancer mortality for the ten most common cancers treated at the University of Kansas Medical Center. Our analysis made use of inverse probability of treatment weighting to balance the sample in terms of sex, age, race, insurance status, marriage status, class of case, and whether this was the first malignancy with respect to rural and urban residents. We selected these factors because they are generally nonmodifiable in health interventions. Furthermore, weighted Cox proportional hazards models were used to address non-proportional hazards across our study time period, providing more reliable estimates of effect. We aimed to consider differences in cancer outcomes among rural vs. urban residences to highlight the need for consideration of local factors related to rural or urban residence for which we can develop outreach, screening, and education interventions to reduce cancer burden. In the population served by the University of Kansas Medical Center, we observed disparities according to rurality that reflect a complex relationship with cancer outcomes, with increased mortality for colon cancer and non-Hodgkin’s lymphoma – nodal tumors as well as decreased mortality for lung cancer compared to urban residents. Our findings emphasize careful consideration of the definition of rural residence, as disparities by rurality likely reflect differential access to care, lifestyle, environmental, and socioeconomic constraints across distinct communities. Our study demonstrates the need for a nuanced view of rural vs. urban cancer outcome disparities and the importance of studies conducted at the local level.

## Supplementary Information

**Additional file 1.** Supplementary Material.

## Data Availability

The datasets generated and/or analyzed during the current study are not publicly available and cannot be shared due to the sensitive nature of the data and restrictions on their availability.

## Abbreviations

C3OD: Curated cancer clinical outcomes database
IPTW: Inverse probability of treatment weighting
KUMC: University of Kansas Medical Center
MHI: Median household income
NHL: Non-Hodgkin’s lymphoma
SAIPE: Small area income and poverty estimates
UIC: Urban influence code
