# Supplementary material for: The need to study rural cancer outcome disparities at the local level: a retrospective cohort study in Kansas and Missouri
Source: BMC Public Health. 2021 Nov 24;21:2154. doi: 10.1186/s12889-021-12190-w (PMC8611913; doi:10.1186/s12889-021-12190-w)
Supplement: Supplementary file 1 — Additional file 1. Supplementary Material. [file 12889_2021_12190_MOESM1_ESM.docx]

Supplementary Material

*Table S1: Cancer Site Recodes*

| **Cancer** | **Site Recodes** |
| --- | --- |
| Breast | 26000 |
| Lung | 22030 |
| Prostate | 28010 |
| Melanoma | 25010 |
| Oral | 20010, 20020, 20030, 20040, 20050, 20060, 20070, 20080, 20090, 20100 |
| Kidney | 29020 |
| Colon | 21041, 21042, 21043, 21044, 21045, 21046, 21047, 21048, 21049 |
| NHL - nodal | 33041 |
| Bladder | 29010 |
| Corpus uteri | 27020 |

*Table S2: Results from IPTW cumulative link models.*

| **Cancer** | **OR** | **95% CI** | **p-value** |
| --- | --- | --- | --- |
| Breast | 1.319 | [1.233, 1.412] | <0.001 |
| Lung | 0.863 | [0.789, 0.945] | 0.001 |
| Prostate | 0.800 | [0.715, 0.895] | <0.001 |
| Melanoma | 1.090 | [0.943, 1.260] | 0.243 |
| Oral | 0.879 | [0.763, 1.014] | 0.077 |
| Kidney | 0.856 | [0.734, 0.996] | 0.045 |
| Colon | 1.299 | [1.124, 1.502] | <0.001 |
| NHL - nodal | 0.886 | [0.754, 1.040] | 0.140 |
| Bladder | 1.241 | [1.036, 1.486] | 0.019 |
| Corpus uteri | 1.113 | [0.909, 1.364] | 0.298 |

*Table S3: Results from unweighted cumulative link models.*

| **Cancer** | **OR** | **95% CI** | **p-value** |
| --- | --- | --- | --- |
| Breast | 1.362 | [1.144, 1.619] | <0.001 |
| Lung | 0.901 | [0.720, 1.128] | 0.360 |
| Prostate | 0.807 | [0.666, 0.979] | 0.030 |
| Melanoma | 1.245 | [0.931, 1.659] | 0.136 |
| Oral | 0.840 | [0.651, 1.087] | 0.183 |
| Kidney | 0.914 | [0.701, 1.188] | 0.506 |
| Colon | 1.149 | [0.836, 1.582] | 0.393 |
| NHL - nodal | 0.968 | [0.676, 1.394] | 0.862 |
| Bladder | 1.270 | [0.896, 1.792] | 0.176 |
| Corpus uteri | 1.278 | [0.835, 1.921] | 0.247 |

*Table S4: Results from IPTW cumulative link models stratified by gender.*

| **Cancer** | **Gender** | **OR** | **95% CI** | **p-value** |
| --- | --- | --- | --- | --- |
| Breast | Female | 1.319 | [1.233, 1.412] | <0.001 |
| Lung | Female | 0.855 | [0.752, 0.972] | 0.017 |
|  | Male | 0.854 | [0.752, 0.969] | 0.014 |
| Prostate | Male | 0.800 | [0.715, 0.895] | <0.001 |
| Melanoma | Female | 0.671 | [0.522, 0.861] | 0.002 |
|  | Male | 1.414 | [1.181, 1.694] | <0.001 |
| Oral | Female | 0.802 | [0.620, 1.037] | 0.093 |
|  | Male | 0.865 | [0.727, 1.029] | 0.102 |
| Kidney | Female | 0.770 | [0.593, 0.998] | 0.049 |
|  | Male | 0.942 | [0.780, 1.138] | 0.536 |
| Colon | Female | 0.994 | [0.811, 1.218] | 0.951 |
|  | Male | 1.589 | [1.292, 1.955] | <0.001 |
| NHL - nodal | Female | 0.581 | [0.452, 0.748] | <0.001 |
|  | Male | 1.111 | [0.896, 1.378] | 0.336 |
| Bladder | Female | 1.285 | [0.899, 1.839] | 0.169 |
|  | Male | 1.192 | [0.967, 1.471] | 0.101 |
| Corpus uteri | Female | 1.113 | [0.909, 1.364] | 0.298 |

*Table S5: Results from unweighted cumulative link models stratified by gender.*

| **Cancer** | **Gender** | **OR** | **95% CI** | **p-value** |
| --- | --- | --- | --- | --- |
| Breast | Female | 1.362 | [1.144, 1.619] | <0.001 |
| Lung | Female | 1.006 | [0.733, 1.383] | 0.972 |
|  | Male | 0.808 | [0.589, 1.112] | 0.189 |
| Prostate | Male | 0.807 | [0.666, 0.979] | 0.030 |
| Melanoma | Female | 0.751 | [0.445, 1.233] | 0.270 |
|  | Male | 1.670 | [1.165, 2.388] | 0.005 |
| Oral | Female | 0.880 | [0.561, 1.382] | 0.577 |
|  | Male | 0.832 | [0.609, 1.143] | 0.252 |
| Kidney | Female | 0.827 | [0.518, 1.299] | 0.418 |
|  | Male | 0.954 | [0.689, 1.316] | 0.777 |
| Colon | Female | 1.116 | [0.698, 1.794] | 0.647 |
|  | Male | 0.186 | [0.769, 1.834] | 0.442 |
| NHL - nodal | Female | 0.738 | [0.405, 1.357] | 0.324 |
|  | Male | 1.078 | [0.687, 1.709] | 0.746 |
| Bladder | Female | 1.302 | [0.657, 2.560] | 0.445 |
|  | Male | 1.258 | [0.837, 1.878] | 0.265 |
| Corpus uteri | Female | 1.278 | [0.835, 1.921] | 0.247 |

*Table S6: Results from weighted Cox proportional hazards models, with IPTW case weighting.*

| **Cancer** | **Avg. HR** | **95% CI** | **p-value** |
| --- | --- | --- | --- |
| Breast | 1.266 | [0.912, 1.759] | 0.159 |
| Lung | 0.823 | [0.694, 0.976] | 0.025 |
| Prostate | 0.780 | [0.487, 1.252] | 0.304 |
| Melanoma | 1.217 | [0.840, 1.762] | 0.299 |
| Oral | 1.118 | [0.873, 1.431] | 0.377 |
| Kidney | 0.876 | [0.655, 1.172] | 0.373 |
| Colon | 1.433 | [1.079, 1.904] | 0.013 |
| NHL - nodal | 1.538 | [1.109, 2.133] | 0.010 |
| Bladder | 1.169 | [0.869, 1.573] | 0.302 |
| Corpus uteri | 1.271 | [0.829, 1.948] | 0.272 |

*Table S7: Results from unweighted Cox proportional hazards models, without IPTW case weighting.*

| **Cancer** | **Avg. HR** | **95% CI** | **p-value** |
| --- | --- | --- | --- |
| Breast | 1.182 | [0.897, 1.558] | 0.234 |
| Lung | 0.857 | [0.743, 0.987] | 0.033 |
| Prostate | 0.538 | [0.353, 0.819] | 0.004 |
| Melanoma | 1.190 | [0.831, 1.706] | 0.343 |
| Oral | 1.196 | [0.947, 1.511] | 0.133 |
| Kidney | 0.931 | [0.708, 1.225] | 0.611 |
| Colon | 1.310 | [1.011, 1.698] | 0.041 |
| NHL nodal | 1.394 | [1.021, 1.904] | 0.037 |
| Bladder | 1.163 | [0.876, 1.542] | 0.296 |
| Corpus uteri | 1.193 | [0.788, 1.805] | 0.404 |

*Table S8: Results from weighted Cox proportional hazards models, with IPTW case weighting stratified by gender.*

| **Cancer** | **Gender** | **OR** | **95% CI** | **p-value** |
| --- | --- | --- | --- | --- |
| Breast | Female | 1.266 | [0.912, 1.759] | 0.159 |
| Lung | Female | 0.838 | [0.652, 1.077] | 0.167 |
|  | Male | 0.813 | [0.650, 1.016] | 0.068 |
| Prostate | Male | 0.780 | [0.487, 1.252] | 0.304 |
| Melanoma | Female | 1.285 | [0.622, 2.652] | 0.498 |
|  | Male | 1.230 | [0.798, 1.894] | 0.348 |
| Oral | Female | 0.809 | [0.503, 1.302] | 0.383 |
|  | Male | 1.281 | [0.963, 1.703] | 0.089 |
| Kidney | Female | 0.898 | [0.544, 1.482] | 0.674 |
|  | Male | 0.849 | [0.595, 1.212] | 0.368 |
| Colon | Female | 1.267 | [0.824, 1.948] | 0.280 |
|  | Male | 1.855 | [1.266, 2.719] | 0.002 |
| NHL - nodal | Female | 2.057 | [1.260, 3.360] | 0.004 |
|  | Male | 1.291 | [0.836, 1.993] | 0.250 |
| Bladder | Female | 1.466 | [0.799, 2.690] | 0.217 |
|  | Male | 1.050 | [0.746, 1.478] | 0.778 |
| Corpus uteri | Female | 1.271 | [0.829, 1.948] | 0.272 |

*Table S9: Results from weighted Cox proportional hazards models, without IPTW case weighting stratified by gender.*

| **Cancer** | **Gender** | **OR** | **95% CI** | **p-value** |
| --- | --- | --- | --- | --- |
| Breast | Female | 1.182 | [0.897, 1.558] | 0.234 |
| Lung | Female | 0.883 | [0.723, 1.078] | 0.221 |
|  | Male | 0.858 | [0.701, 1.052] | 0.140 |
| Prostate | Male | 0.538 | [0.353, 0.819] | 0.004 |
| Melanoma | Female | 0.967 | [0.477, 1.957] | 0.925 |
|  | Male | 1.294 | [0.851, 1.967] | 0.229 |
| Oral | Female | 0.766 | [0.485, 1.209] | 0.252 |
|  | Male | 1.421 | [1.081, 1.868] | 0.012 |
| Kidney | Female | 0.952 | [0.591, 1.534] | 0.840 |
|  | Male | 0.905 | [0.650, 1.260] | 0.555 |
| Colon | Female | 1.235 | [0.843, 1.809] | 0.279 |
|  | Male | 1.425 | [1.007, 2.016] | 0.046 |
| NHL - nodal | Female | 1.837 | [1.131, 2.984] | 0.014 |
|  | Male | 1.129 | [0.751, 1.697] | 0.561 |
| Bladder | Female | 1.557 | [0.877, 2.765] | 0.130 |
|  | Male | 1.048 | [0.759, 1.448] | 0.776 |
| Corpus uteri | Female | 1.193 | [0.788, 1.805] | 0.404 |


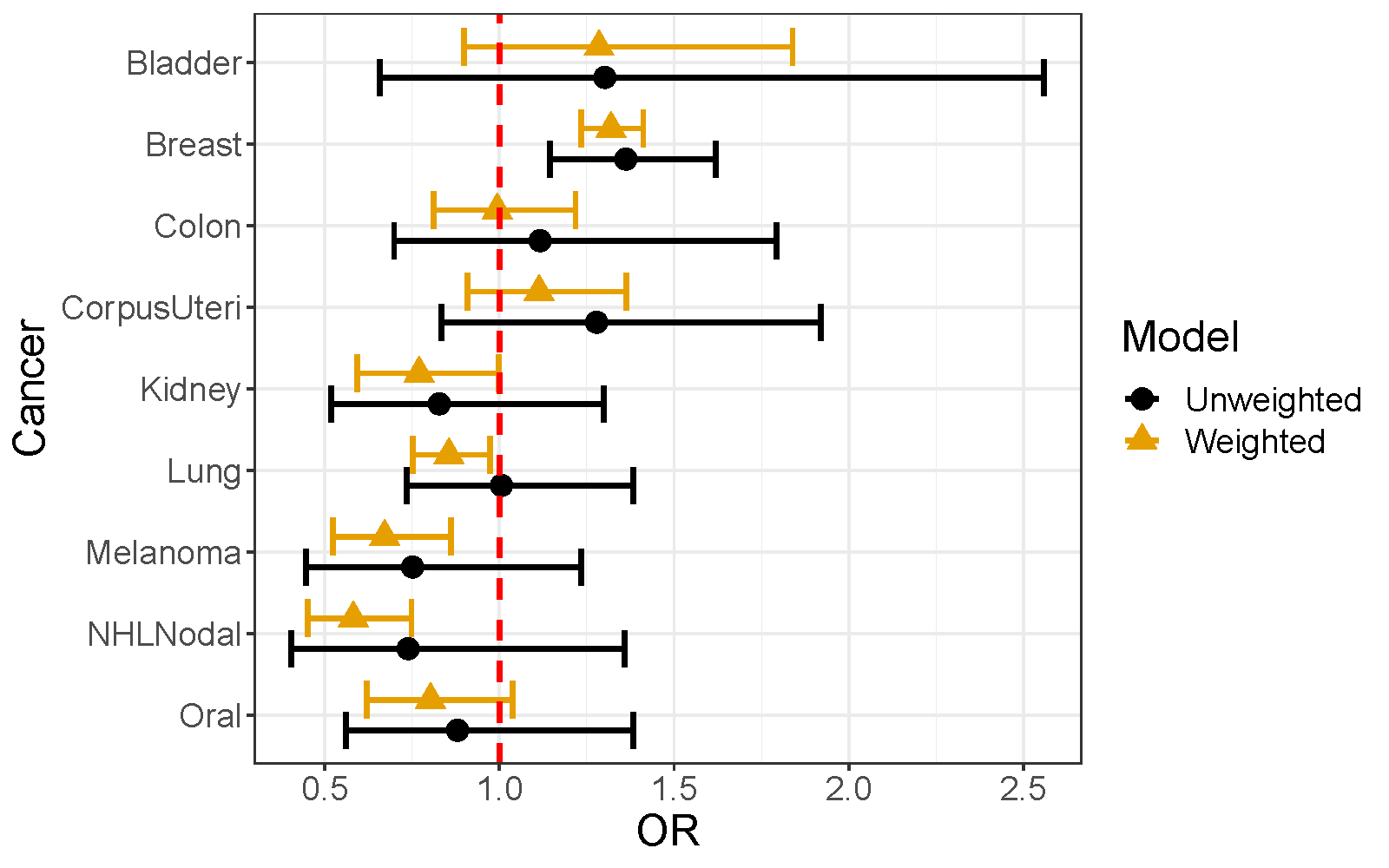


Figure S1: Cancer stage in rural vs. urban female residents. The figure shows odds-ratios, with 95% confidence intervals, for the odds of being at a higher stage for rural vs. urban female residents. We show results for both IPTW weighted and unweighted models. For the IPTW weighted models, rural residence had a significant effect on stage for breast, kidney, lung, and melanoma in females. Rural residency was associated with increased stage in breast but decreased stage for kidney, lung, and melanoma.


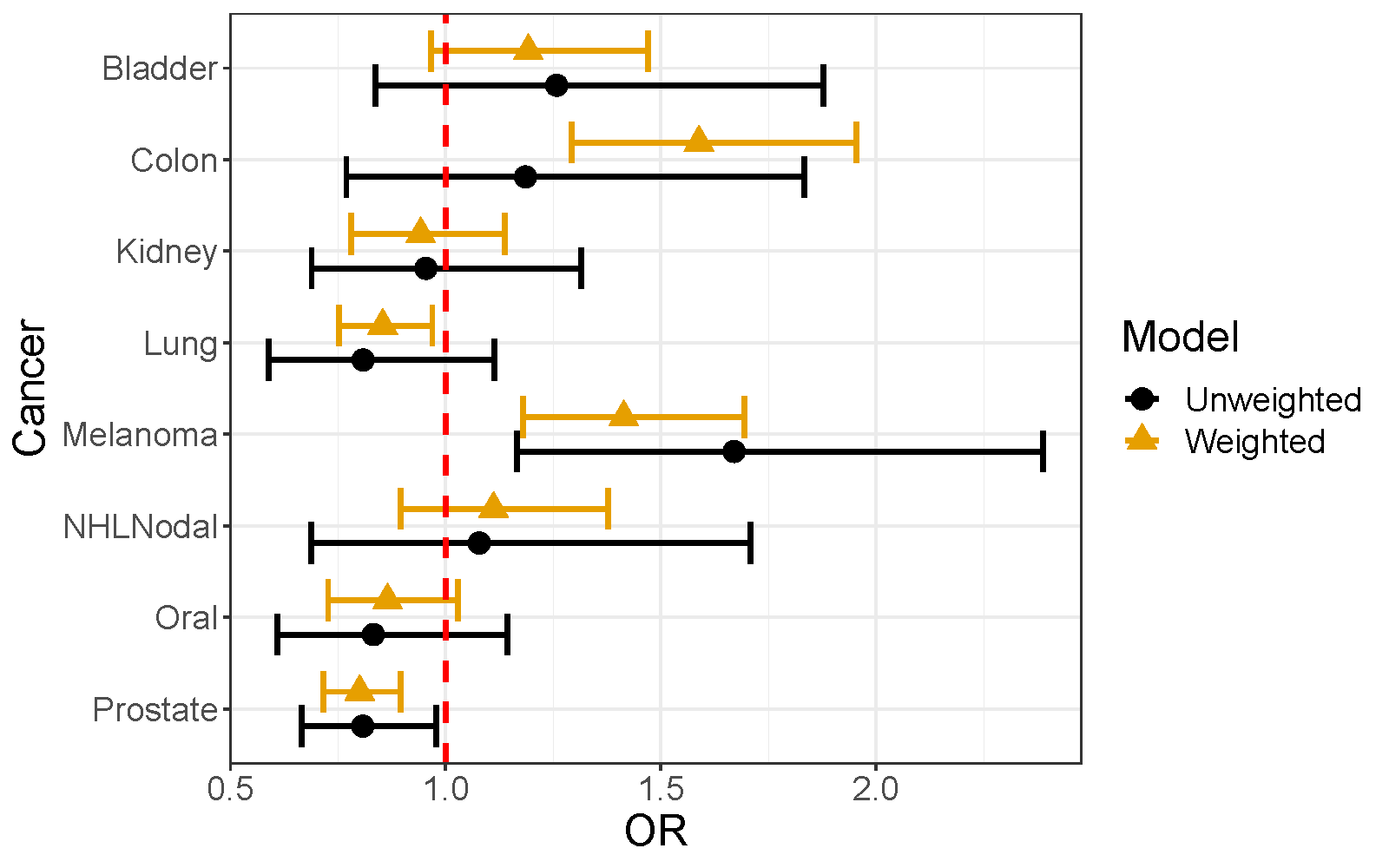


Figure S2: Cancer stage in rural vs. urban male residents. The figure shows odds-ratios, with 95% confidence intervals, for the odds of being at a higher stage for rural vs. urban male residents. We show results for both IPTW weighted and unweighted models. For the IPTW weighted models, rural residence had a significant effect on stage for colon, lung, and melanoma in males. Rural residency was associated with increased stage in colon and melanoma but decreased stage for lung cancer.


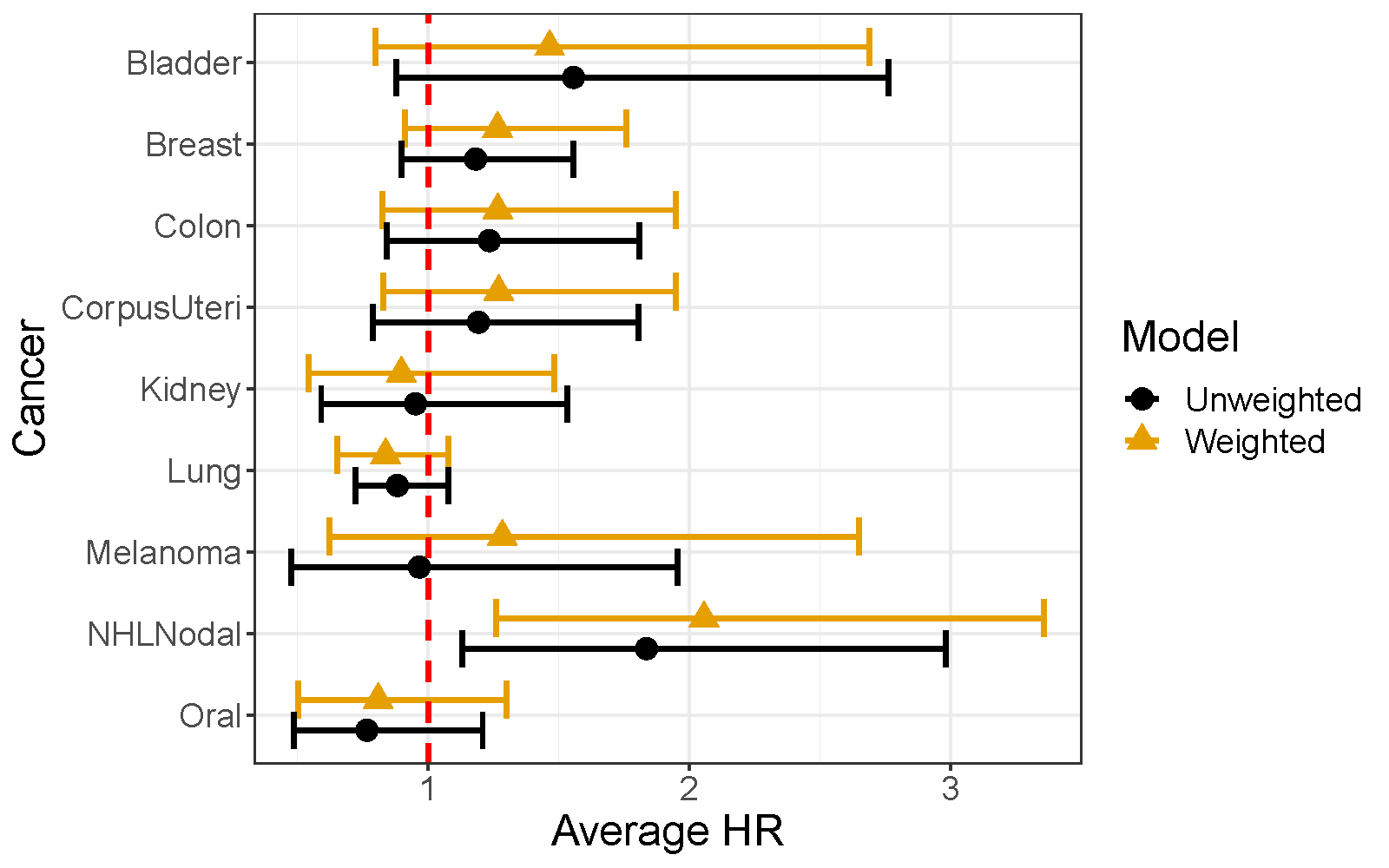


Figure S3: Hazard ratios for rural vs. urban female residents. The figure shows average hazard-ratios, with 95% confidence intervals, for the hazards of rural vs. urban female residents with respect to overall survival. We show results for both IPTW weighted and unweighted models. For the IPTW weighted models, rural residence was associated with significantly increased hazards for females with non-Hodgkin’s lymphoma.


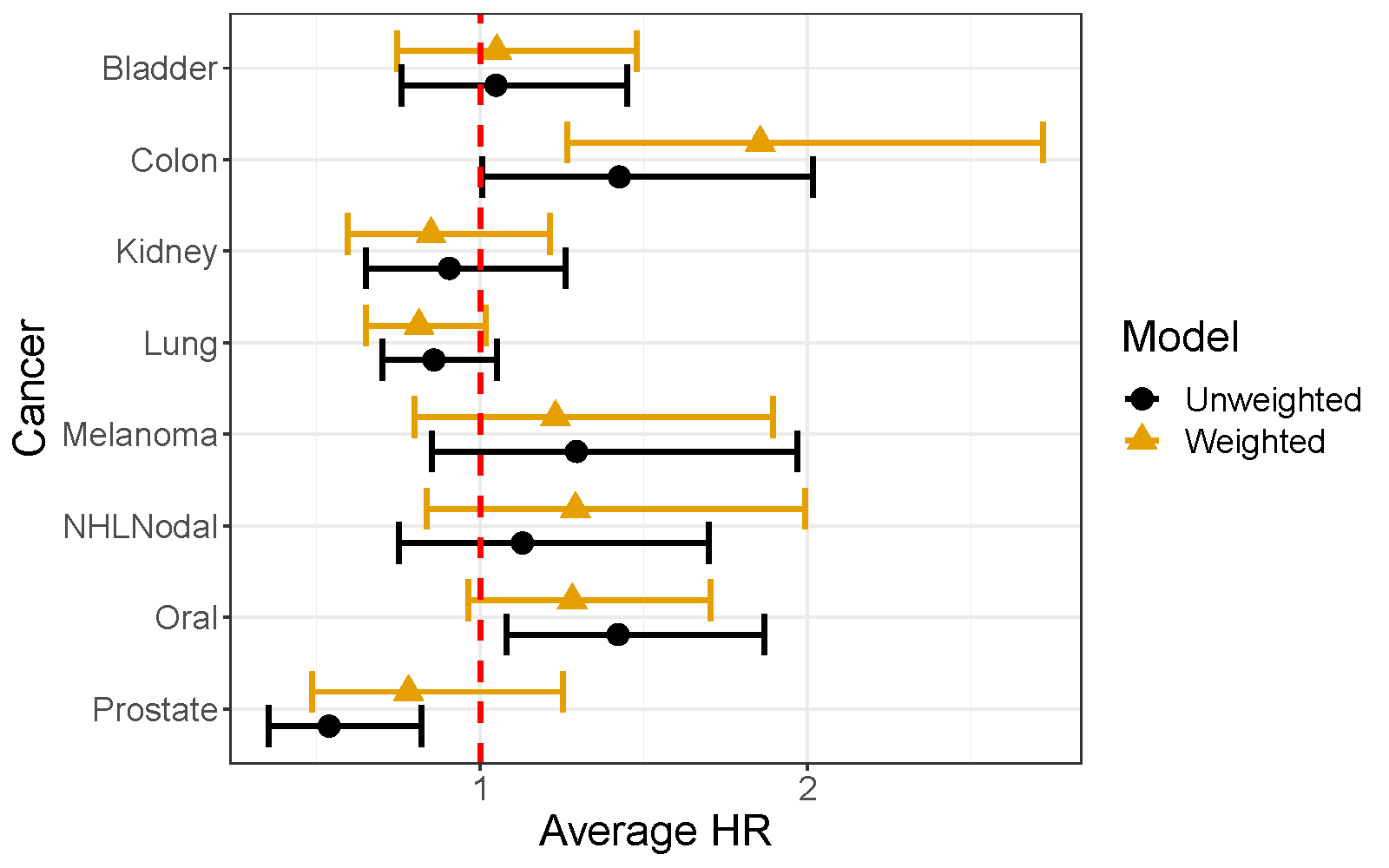


Figure S4: Hazard ratios for rural vs. urban male residents. The figure shows average hazard-ratios, with 95% confidence intervals, for the hazards of rural vs. urban male residents with respect to overall survival. We show results for both IPTW weighted and unweighted models. For the IPTW weighted models, rural residence was associated with significantly increased hazards for males with colon cancer.
